# Supplementary material for: MetaRibo-Seq measures translation in microbiomes
Source: Nat Commun. 2020 Jun 29;11:3268. doi: 10.1038/s41467-020-17081-z (PMC7324362; doi:10.1038/s41467-020-17081-z)
Supplement: Supplementary file 10 — Supplementary Data 7 [file 41467_2020_17081_MOESM10_ESM.zip › File2/Confidence_VeryHigh_Taxonomy/260543_out.krona.html]

Javascript must be enabled to view this page.

members
magnitude
magnitudeUnassigned
count
unassigned
taxon
rank

260543\_out

16

superkingdom
2
16

16
1239
phylum

1
class
91061

1
186826
order

1
family
1300

1
1301
genus

1

SRS893273\_contig\_number\_6823
species
2069309

class
186801
15

15
order
186802

family
543314
5


SRS012279\_contig\_number\_20277SRS015434\_contig\_number\_14380SRS019071\_contig\_number\_33651SRS022077\_contig\_number\_17412SRS1054712\_contig\_number\_445
species
143393
5

186803
family
10

genus
572511
10

33038

SRS014736\_contig\_number\_9033SRS014736\_contig\_number\_9033SRS014736\_contig\_number\_9033SRS014948\_contig\_number\_10082SRS014948\_contig\_number\_10082SRS014948\_contig\_number\_10082SRS022137\_contig\_number\_21639SRS048060\_contig\_number\_12094SRS048060\_contig\_number\_12094SRS048870\_contig\_number\_22688
species
10
